# Bone Cuts Accuracy of a System for Total Knee Arthroplasty including an Active Robotic Arm

**DOI:** 10.3390/jcm10163714

**Published:** 2021-08-20

**Authors:** Killian Cosendey, Julien Stanovici, Jaad Mahlouly, Patrick Omoumi, Brigitte M. Jolles, Julien Favre

**Affiliations:** 1 Swiss BioMotion Lab, Department of Musculoskeletal Medicine, Lausanne University Hospital and University of Lausanne (CHUV-UNIL), CH-1011 Lausanne, Switzerland; julien.stanovici@chuv.ch (J.S.); jaad.mahlouly@chuv.ch (J.M.); brigitte.jolles-haeberli@chuv.ch (B.M.J.); julien.favre@chuv.ch (J.F.); 2 Service of Diagnostic and Interventional Radiology, Lausanne University Hospital and University of Lausanne (CHUV-UNIL), CH-1011 Lausanne, Switzerland; patrick.omoumi@chuv.ch; 3 Institute of Microengineering, Ecole Polytechnique Fédérale Lausanne (EPFL), CH-1015 Lausanne, Switzerland

**Keywords:** total knee arthroplasty, robotic-assisted surgery, position and orientation error, registration, level of experience

## Abstract

Introduction: This study aimed to assess the bone cuts accuracy of a system for total knee arthroplasty including an active robotic arm. A second objective was to compare the accuracy among orthopaedic surgeons of different levels of experience. Methods: Three orthopaedic surgeons cut 10 sawbone knees each. Planned and actual bone cuts were compared using computed tomography. Difference with respect to the planning was expressed as three position and three orientation errors following the anatomical planes. Statistical tests were performed to detect bias and compare surgeons. Results: None of the 30 knees presented an outlier error, meaning an error ≥3 mm or ≥3°. The root-mean-square values of the 12 error types were below 0.8 mm or 0.8°, except for the femoral proximal–distal errors (1.7 mm) and the tibial anterior-posterior errors (1.4 mm). Biases were observed, particularly in femoral proximal–distal and tibial anterior–posterior positions. Median differences between surgeons were all lower than 0.8 mm and 0.5°, with statistically significant differences among surgeons in the femoral proximal–distal errors and the tibial anterior–posterior errors. Conclusions: The system tested in this study achieved accurate bone cuts independently of the surgeon’s level of experience. Biases were observed, suggesting that there might be options to improve the accuracy, particularly in proximal–distal position for the femur and in anterior–posterior position for the tibia.

## 1. Introduction

The number of Total Knee Arthroplasty (TKA) procedures performed yearly is continuously increasing worldwide. For example, in the United States of America, an increase of 143% is expected between 2012 (631,000 cases) and 2050 (1,532,000 cases) [1]. Robotics offers interesting perspectives to help to manage this increasing surgical demand. In fact, robotic-assisted TKA (RATKA) could improve implant placement and mechanical alignment accuracy [2,3], two elements associated with implant survival and patient quality of life [3,4,5]. Moreover, RATKA could diminish the influence of the surgeon’s level of experience [6], with less experienced surgeons achieving comparable accuracy than more experienced surgeons. While promising, RATKA remains an emerging technology and there is a need to extend the characterization of current solutions to ensure their optimal uses and suggest possible areas of enhancement.

The *TSolution One^®^ Total Knee Application* (“*TSOLUTION ONE” in this article,* THINK Surgical Inc., Fremont, CA, USA) is a particularly interesting RATKA option because it includes an active robotic arm performing the bone cuts autonomously. The early version of this system (*ROBODOC^®^*) was shown to improve mechanical alignment by decreasing the percentage of outliers (i.e., knees differing by 3° or more from the target), in comparison to conventional methods [7,8]. A decrease in the percentage of outliers was also reported for the orientation of the femoral and tibial implants in the sagittal and coronal planes [7,9]. While helpful, these studies characterized the earlier version of the system and literature is missing regarding *TSOLUTION ONE* accuracy. In addition, prior studies did not conduct full assessments, meaning that the orientation accuracy in the axial plane as well as the position accuracy in the three anatomical planes have not been reported. Since these characteristics [10,11], as well as the ones assessed on the early version [7,8,9], could be important factors of surgical success, there remains a need to assess the accuracy of *TSOLUTION ONE* in all six degrees-of-freedom for the femur and tibia. Since no study has yet reported the variations in bone cuts accuracy among surgeons, there is an interest in evaluating *TSOLUTION ONE* with surgeons of different levels of expertise.

Therefore, the purpose of this study was to assess the accuracy of the bone cuts done by *TSOLUTION ONE* in terms of position and orientation. This study also aimed to compare the accuracy among orthopaedic surgeons of different levels of experience. To limit the confounding factors arising from the natural inter-individual variability, such as varying bone size or mineral density, this study was performed using sawbones.

## 2. Materials and Methods

### 2.1. Experimental Setup

Three orthopaedic surgeons from the same university hospital with different levels of experience participated in this study: one “highly experienced” surgeon with more than 15 years of independent total knee arthroplasty practice, one “experienced” surgeon with approximatively 5 years of independent total knee arthroplasty practice and one “fellow” surgeon not yet performing total knee arthroplasty independently. They were trained on *TSOLUTION ONE* (version 300), including its planning workstation (*TPLAN*^®^) and its robotic-assisted surgery device (*TCAT*^®^), before preforming the cases analysed in this study. None had prior experience in RATKA. For this study, each surgeon planned and cut 10 right sawbone knees, following the manufacturer’s recommendations. The six-step protocol used for the 30 knees in this study is described below.

Step 1: 10 fiducial markers (metallic beads of 0.8 mm diameter) were embedded in the femur and tibia sawbones (Pacific Research Company, Vashon Island, WA, USA) to allow registering the original sawbones (before cutting) with the cut sawbones in step 4.

Step 2: The original sawbones were CT scanned using a Discovery CT750 HD machine (GE Healthcare, Chicago, Illinois) parametrized as follows: field of view of 250 × 250 mm, matrix size of 512 × 512 pixels, tube voltage of 120 kVp and tube current of 200 mAs. Two set of images were extracted with slice thicknesses of 0.312 mm (high resolution) and 0.625 mm (low resolution), respectively. The lower resolution images were uploaded in the planning software in order to reconstruct the 3D surface model of the sawbones and plan the arthroplasty. For this study, all procedures were planned with the “Persona Posterior Stabilized Standard”, size 8, femoral component and the “Persona Posterior Stabilized Keel”, size D, tibial component (Zimmer, Warsaw, IN, USA). Once completed, the planning was uploaded in the robotic device.

**Figure 1:**
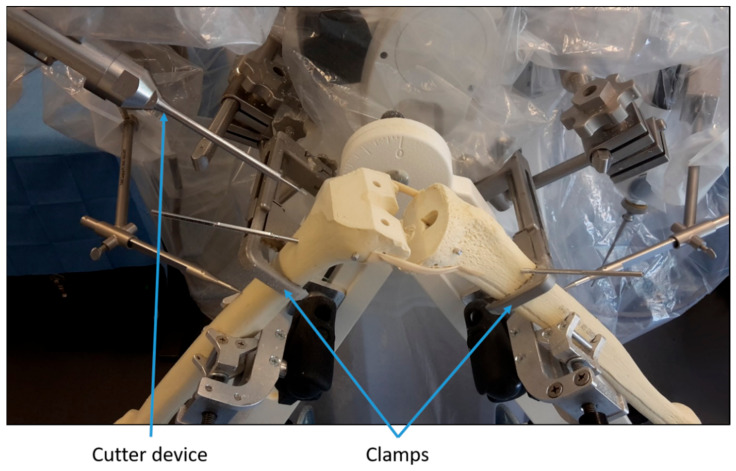
Illustration of the experimental setup.

Step 3: The robotic device was prepared and calibrated as for any intervention on a patient. Next, the sawbones were positioned similarly to a real procedure and fixed with clamps (Figure 1). Then, the position of registration points on the surface of the sawbones were recorded by the surgeons using a mechanical digitizer. The surgeons were guided by instructions on a screen during this step. Once the points were recorded, the algorithms within the robotic device registered the virtual bones from the planning to the sawbones. After that, the robot milled the femoral and tibial sawbones under the surgeons’ supervision (Figure 1).

Step 4: The cut sawbones were CT scanned using the same high resolution parameters as for the initial scanning. The images obtained that way were segmented using in-house software to reconstruct the 3D surface model of the cut sawbones [12,13]. Then, the models of the original and cut sawbones were imported in Matlab (Mathworks, Natick, MA, USA) and registered using the fiducial markers. Specifically, separately for the tibia and the femur, the registration was completed by locating the centre of the markers and calculating the mathematical transformation mapping the markers of the cut sawbone to the markers of the original sawbone [14].

Step 5: To quantify the accuracy, a reference frame was defined for the femoral cut (*XYZ_femur_*) and another one for the tibial cut (*XYZ_tibia_*), based on the shape of the cuts. Then, using the registered data, these frames were located for the planning on the original sawbones (*XYZ^planning^*) and for the actual cuts (*XYZ^cut^*). Next, the difference between the planning and cut frames were calculated for the femur ($XYZ_{femur}^{planning}$ vs. $XYZ_{femur}^{cut}$) and the tibia ($XYZ_{tibia}^{planning}$ vs. $XYZ_{tibia}^{cut}$) of each knee. Finally, for each bone, the difference between the planning and cut frames was expressed as three position and three orientation errors [15]. By convention, positive femoral errors indicate a cut too anterior, proximal, lateral, varus, internally rotated and extended compared to the planning. Positive tibial errors have similar meanings to positive femoral errors, except in the coronal and sagittal planes, where positives errors indicate a cut too valgus and flexed compared to the planning.

To assess the repeatability of the error measurement method, the cut sawbones of two randomly-selected knees were CT scanned and processed five times each. This procedure indicated root-mean-square (RMS) repeatability between 0.03 mm and 0.09 mm for all position errors. The repeatability of the orientation errors were between 0.09° and 0.18°, except for femoral flexion–extension (0.39°) and tibial internal–external rotation (0.51°).

### 2.2. Statistical Analysis

Since the errors followed non-normal distributions, they were reported through their median, interquartile range (IQR) and root-mean-square (RMS). These statistics were calculated for the pooled errors of the three surgeons (*n* = 30 per type of error) and for each surgeon independently (*n* = 10 per type of error). Wilcoxon signed-rank tests were performed to detect errors different from zero, therefore indicating biases in the cuts. Kruskal–Wallis tests, with post-hoc Wilcoxon rank-sum tests, were conducted to compare the errors among the three surgeons. The significance level was set a priori to 5%. In addition to the continuous characterization of the errors above, the percentage of outliers was calculated for each type of error, considering the usual definition of an outlier being a knee with a difference between the planning and the cut outside the +/−3 mm or +/−3° range [10,16,17].

## 3. Results

### 3.1. Bone Cut Accuracy Using the Robotic-Assisted System

For the 30 knees in this study, the RMS values of all errors were below 0.8 mm or 0.8°, except for the femoral proximal–distal errors (1.7 mm) and the tibial anterior–posterior errors (1.4 mm) (Table 1). The variability (IQR) among the 30 knees was between 0.4 to 0.6 mm for position errors and between 0.2 and 1.0° for orientation errors. Biases were observed for 8 of the 12 error types assessed in this study. In fact, in median, the femoral cuts were systematically too anterior by 0.3 mm (*p* < 0.01), proximal by 1.6 mm (*p* < 0.01), and lateral by 0.8 mm (*p* < 0.01), compared to the planning. For the tibia, in median, the cuts were systematically too posterior by 1.4 mm (*p* < 0.01), distal by 0.4 mm (*p* < 0.01), lateral by 0.6 mm (*p* < 0.01), valgus by 0.5° (*p* < 0.01) and flexed by 0.2° (*p* = 0.03), compared to the planning. The magnitude of the biases exceeded the repeatability of the error measurement method by at least six-fold, except for the flexion–extension of the tibia where the magnitude of the bias was 1.3 times larger than the repeatability of the measurement method. There was no outlier, neither for femoral nor tibial errors.

### 3.2. Influence of the Surgeon’s Level of Experience

**Figure 2:**
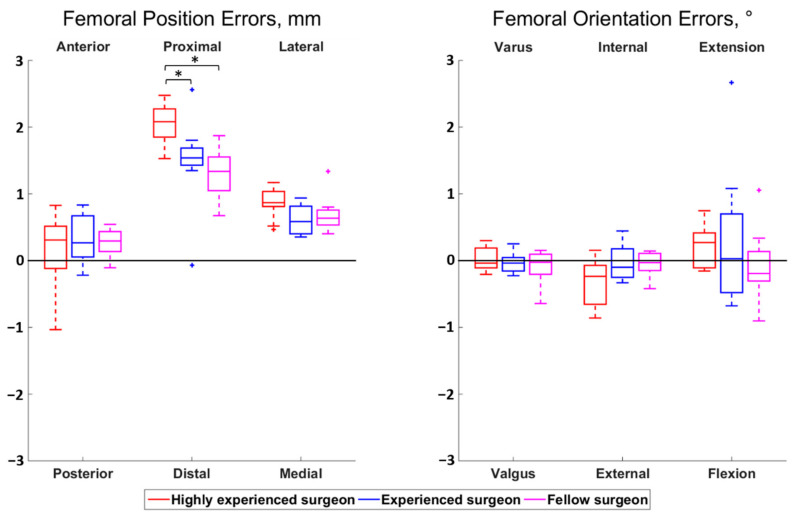
Boxplot of the position (**left**) and orientation (**right**) errors for the femoral cut. The direction at the top and bottom of the plots indicate where the actual cuts were compared to the planned cuts. For example, a positive anterior–posterior error indicates a cut that was too anterior compared to the planning. Statistically significant differences among surgeons are asterisked (*p* < 0.05).

**Figure 3:**
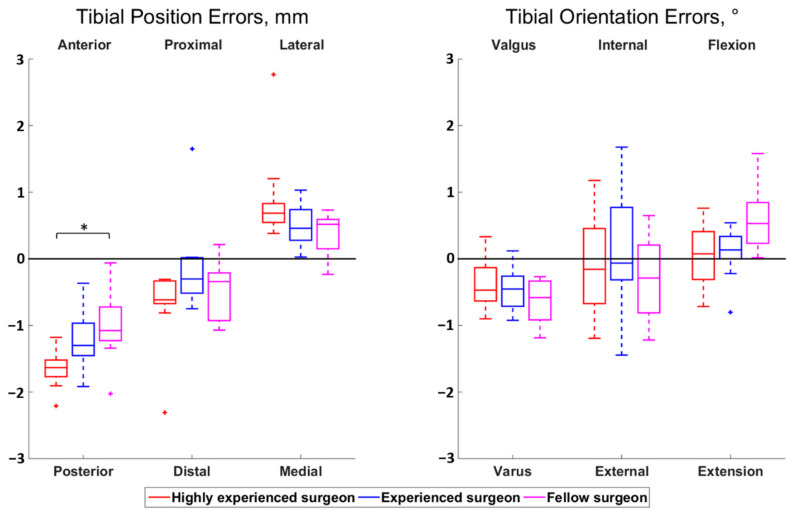
Boxplot of the position (**left**) and orientation (**right**) errors for the tibial cut. The direction at the top and bottom of the plots indicate where the actual cuts were compared to the planned cuts. For example, a positive anterior–posterior error indicates a cut that was too anterior compared to the planning. Statistically significant differences among surgeons are asterisked (*p* < 0.05).

The magnitudes of all median surgeon-to-surgeon differences were below 0.8 mm for the position errors and below 0.5° for the orientation errors. Statistically significant differences among surgeons were observed for the femoral proximal–distal errors (*p* < 0.01) and the tibial anterior–posterior errors (*p* = 0.01) (Figure 2 and Figure 3). Regarding the femur, the cuts performed by the highly experienced surgeon were more proximal than the cuts performed by the experienced (*p* = 0.02) and fellow (*p* < 0.01) surgeons, in median, by 0.5 mm and 0.7 mm, respectively. For the tibia, the cuts from the highly experienced surgeon were more posterior than the cuts from the fellow surgeon (*p* = 0.01), in median, by 0.6 mm.

## 4. Discussion

The RATKA system assessed in this study achieved accurate bone cuts as none of the 360 errors (30 knees × 12 types of error) exceeded the usual +/−3 mm or +/−3° thresholds defining outlier cuts [17,18]. Furthermore, 10 out of the 12 types of errors had RMS values lower than 1 mm or 1°. Nevertheless, biases were observed, particularly for the two types of errors with larger RMS values (femoral proximal–distal position (1.6 mm) and tibial anterior-posterior position (1.4 mm)). Since the variations among knees were small (IQR in the range 0.3 to 0.6 mm or 0.2 and 1.0°), there might be options to diminish the constituent of the errors common to all knees and thus improve the accuracy, especially for the two errors types with larger biases. Further research will be necessary to assess this possibility and determine its clinical impact, given that there is a paucity of literature regarding the effects of small changes in bone cuts accuracy on clinical outcomes [19].

To the authors’ knowledge, only two studies on RATKA bone cuts accuracy have been published so far; none evaluating all six degrees-of-freedom. One of them tested a semiautonomous system using 30 cadaveric knees and reported errors with RMS values ranging from 0.5 to 1.3° and standard deviations (SD) ranging from 0.5 to 0.9° [20]. Additionally, this previous study reported between 0 and 3% of outliers. The other prior study on RATKA bone cuts accuracy also assessed a semiautonomous system, this time using 6 cadaveric knees [21]. Median errors between 0.3 and 1.1° (SD between 0.1 and 1.0°) were reported. Although these prior works and the present study differ on many experimental aspects (e.g., robotic approach, number and condition of the test knees, and assessment methods), the results are rather consistent, showing the potential of RATKA to achieve accurate bone cuts. Looking at the errors that are comparable between the two prior works and the present study tends to indicate lower errors in the present study. However, the differences are small (RMS values smaller by 0.1 to 0.6°, median errors smaller by 0.1 to 0.5°, SD smaller by 0.5° to larger by 0.1°) and further studies with direct comparisons are necessary to determine if one RATKA system outperforms the others.

The magnitudes of the differences among surgeons were all lower than 0.8 mm or 0.5°, suggesting that they are irrelevant clinically [19]. Interestingly, beyond the fact that differences among surgeons are certainly irrelevant clinically, the results suggest that a lower level of experience is not associated with lower bone cuts accuracy when the procedures are performed using the active robotic system tested in this study. This observation is particularly important in view of the increasing demand for total knee arthroplasty worldwide [1]. While further work will be necessary to determine the exact mechanism causing the statistically significant differences among surgeons, the differences are probably due to the manual recording of registration points on the bone surfaces (Step #3 in the Methods section). To our knowledge, this is the first study to assess the influence of the surgeon’s level of experience on the bone cuts accuracy in RATKA, preventing comparison with others robotic options.

Using sawbones in this study provided several advantages. Firstly, it allowed comparing cases and surgeons without confounders associated to the natural variability among individual human knees such as varying bone size or mineral density. Secondly, the possibility to embed metallic beads in the sawbones improved the assessment. Thirdly, the absence of ethical concern when assessing TKA on sawbones made the testing of a large number of knees possible. Lastly, the standardization provided by the use of sawbones will facilitate the comparison between the present results and the results of future studies also conducted with sawbones. On the other hand, there were some limitations related to the use of sawbones. Firstly, the procedures were simplified, as there was no surrounding soft tissue, cartilage, nor bone deformity. Secondly, the constraint to be quick and the mental pressure associated with the accomplishment of a TKA procedure on a patient were absent. These simplifications compared to real cases could influence the error profile. Nevertheless, in view of the study objectives, and since the procedures were guided by the RATKA system, one can assume these simplifications to have negligible effects on the overall findings.

This study focussed on the accuracy of the bone cuts, and future works should assess the accuracy of the entire procedure, including the implantation of the prosthesis components. Not implanting a prosthesis was beneficial in this study because it allowed a clear view of the cut bones in the CT images, without artefact from the metallic components. The small number of surgeons could also be seen as a limitation. However, the number of knees used for the surgeons comparison (*n* = 30 in total) combined with the consistency of the results suggest that the overall findings would have been similar with a larger number of surgeons.

## 5. Conclusions

The RATKA system tested in this study achieved accurate bone cuts independently of the surgeon’s level of experience, both in terms of position and orientation. Biases were nonetheless observed, suggesting that the errors could be reduced. Further research is warranted to compare the six degree-of-freedom accuracy between RATKA systems and determine the relationships between bone cuts accuracy and clinical outcomes.

## Figures and Tables

**Table 1:** Pooled errors of the three surgeons (*n* = 30 knees).

Error Type			Median {IQR}	RMS	Percentage of Outliers	Mean ± SD ^#^
Femur	Position	Anterior error	0.29 {0.45} *	0.45	0	0.25 ± 0.38
		Proximal error	1.62 {0.49} *	1.71	0	1.63 ± 0.55
		Lateral error	0.75 {0.35} *	0.76	0	0.72 ± 0.25
	Orientation	Varus error	−0.04 {0.24}	0.19	0	−0.03 ± 0.19
		Internal rotation error	−0.13 {0.41}	0.32	0	−0.12 ± 0.30
		Extension error	0.03 {0.61}	0.70	0	0.14 ± 0.69
Tibia	Position	Anterior error	−1.35 {0.58} *	1.39	0	−1.30 ± 0.50
		Proximal error	−0.41 {0.40} *	0.73	0	−0.43 ± 0.61
		Lateral error	0.57 {0.38} *	0.78	0	0.60 ± 0.52
	Orientation	Valgus error	−0.49 {0.44} *	0.61	0	−0.50 ± 0.36
		Internal rotation error	−0.15 {0.99}	0.76	0	−0.12 ± 0.77
		Flexion error	0.24 {0.51} *	0.54	0	0.25 ± 0.48

The values in this table indicate where the actual cuts were compared to the planned cuts. For example, a positive anterior error indicates a cut that was too anterior compared to the planning, similarly a negative anterior error indicates a cut that was too posterior compared to the planning. Median, IQR, RMS, mean and SD data are in mm for position errors and in degrees for orientation errors. IQR: interquartile range; RMS: root-mean-square; SD: standard deviation. *: statistically significantly different from zero (*p* ≤ 0.05). ^#^: although data followed non-normal distributions, they were also reported as mean ± SD to allow comparison with prior literature.
